# Polyclonal glycine receptor aAbs: a challenge for personalized epitope characterization

**DOI:** 10.3389/fnmol.2026.1747209

**Published:** 2026-03-17

**Authors:** Anna-Lena Wiessler, Inken Stahl, Natascha Schaefer, Vera Rauschenberger, Ivan Talucci, Hans M. Maric, Betül Baykan, Erdem Tüzün, Claudia Sommer, Carmen Villmann

**Affiliations:** 1 Institute for Clinical Neurobiology, University Hospital Würzburg, Würzburg, Germany; 2 Department of Neurology, University Hospital Würzburg, Würzburg, Germany; 3 Rudolf Virchow Center for Integrative and Translational Bioimaging, University of Würzburg, Würzburg, Germany; 4 Department of Neurology and Clinical Neurophysiology, EMAR Medical Center, Istanbul, Türkiye; 5 Institute of Experimental Medicine, Istanbul University, Istanbul, Türkiye

**Keywords:** autoantibodies, epitope, glycine receptor, neutralization, stiff person syndrome

## Abstract

**Introduction:**

Patients with glycine receptor (GlyR) aAbs suffer from various diseases, including stiff-person syndrome (SPS), and currently, no cure exists. Several treatment options exist; however, these treatment options lack specificity. To date, only one common epitope has been mapped for GlyR aAbs in the far N-terminal region of the GlyRα1 subunit. However, some patient sera also bind GlyRα2, GlyRα3, or GlyRβ. Therefore, more than one common epitope may exist. Unraveling these epitopes will help generate more specific treatment approaches.

**Methods:**

Here, we constructed GlyRa1 and GlyRa3 variants by site-directed mutagenesis using amino acid differences between these two subunits within their extracellular domains. Peptide microarrays, which have shown that an epitope including the binding site of a commercial pan-a antibody (^96^PDLFFANEKS^105^) and its surrounding residues is highly relevant for aAb binding, were utilized to identify additional residues important for aAb binding. Two overlapping peptides (^93^LWKPDLFFANEKSAN^107^ and ^98^LFFANEKSANFHDVT^112^) were used for aAb neutralization in cell-based assays.

**Results:**

The GlyRa1 and GlyRa3 variants helped to identify which amino acid sequences in the extracellular domain of GlyRs represent additional aAb epitopes or are involved in aAb binding. Using both generated peptides for aAb neutralization with a patient serum containing GlyRb aAbs that bind specifically to this region ^96^PDLFFANEKSANFHDV^111^, successful neutralization was demonstrated. In contrast, when using patient sera that reliably target the extracellular domain including ^96^PDLFFANEKS^105^ of the GlyRa subunits, the overlapping peptides reduced aAb binding but failed to fully neutralize the aAbs.

**Discussion:**

In conclusion, our data demonstrate that GlyR aAbs are polyclonal or bind to structural epitopes. These results define single residues important for aAb binding and help explain why no further common aAb binding site has been identified so far. Hence, patient-specific pattern for GlyR aAbs exist, emphasizing the importance of epitope characterization as basis for future therapeutic testing or even complete neutralization of the aAbs.

## 1 Introduction

Glycine receptor (GlyR) autoantibodies (aAbs) in patients have been associated with a variety of clinical syndromes, including stiff person syndrome (SPS), progressive encephalomyelitis with rigidity and myoclonus (PERM) (Carvajal-Gonzalez et al., 2014), and epilepsy and encephalitis (Ekizoglu et al., 2014; Martinez-Hernandez et al., 2016; Swayne et al., 2018; Crisp et al., 2019). The main symptoms in patients with SPS are spasms and stiffness of skeletal muscles (Dalakas, 2008; Enuh et al., 2014; Sabatino and Newsome, 2017).

The target of GlyR aAbs is the pentameric ligand-gated chloride channel. GlyRs form α homomers, mainly located at pre- and extrasynaptic sites, or αβ heteromers at postsynaptic sites (Dutertre et al., 2012). They are expressed in the mammalian spinal cord and brainstem and play an important role in inhibitory signal transmission (Malosio et al., 1991; Dutertre et al., 2012; Weltzien et al., 2012). The described pathomechanisms of GlyR aAbs include complement activation and increased internalization rates of targeted GlyRs, leading to reduced surface expression of the receptor (Carvajal-Gonzalez et al., 2014) as well as direct alterations in ion channel function, leading to decreased inhibitory signal propagation (Crisp et al., 2019; Rauschenberger et al., 2020; Wiessler et al., 2024b; Wiessler et al., 2025). The spasms and stiffness of skeletal muscles observed in patients may result from these functional alterations.

A common aAb binding epitope, ^29^A-^62^G (numbers refer to the immature protein), in the far N-terminal region of the GlyRα1 subunit was described. Furthermore, GlyR glycosylation is non-essential for aAb binding (Rauschenberger et al., 2020; Rauschenberger et al., 2023). Whether there are additional epitopes within this highly conserved extracellular region of GlyR subunits remains to be determined. Other GlyRα subunits (Carvajal-Gonzalez et al., 2014) or the GlyRβ subunits have also been identified as targets of GlyR aAb in some patient samples. Targeting the GlyRβ subunit occurs in different extracellular sequence domains apart from the common far N-terminal epitope estimated in GlyRα1 (Wiessler et al., 2024b). In addition, both pre- and postsynaptic GlyRs, which differ in their subunit compositions, can be targeted by patient aAbs (Wiessler et al., 2025). In recent years, for different types of autoimmune encephalitis with aAbs against, e.g., NMDA receptor, LG1, and GABA_A_ receptor, monoclonal antibodies have been isolated from patients (Kreye et al., 2016; Kornau et al., 2020; Kreye et al., 2021; Noviello et al., 2022). These monoclonal antibodies have tremendously increased our understanding of the molecular mechanisms underlying autoimmune encephalitis. For GlyR aAbs, however, (i) aAb-positive cerebrospinal fluid sample availability is limited, and (ii) isolation of monoclonal antibodies from patient CSF has been, to date, unsuccessful. Epitope mapping and investigations of pathophysiological mechanisms still rely on cell- and tissue-based assays.

Moreover, patients with GlyR aAb-associated diseases exhibit a large variety of clinical presentations and diverse symptoms. The estimated binding patterns of patient aAbs to various regions within the CNS may explain the individual differences in patients’ clinical symptoms (Piro et al., 2025). However, whether different binding patterns can be correlated with functional alterations of distinct molecular correlates is not yet fully understood. The present study investigates the identification of further distinct GlyR aAb binding epitopes, which may help understand the full picture of their pathological mechanisms.

## 2 Materials and methods

### 2.1 Ethical considerations

The use of patient blood samples for experiments was approved by the Ethics Committee of the Medical Faculty of the University of Würzburg, Germany, concerning the project “Glycine receptor autoantibodies and spinal disinhibition” (reference number 20190424 01).

### 2.2 Patients

For this study, we used serum samples from 9 patients, including 7 patients diagnosed with SPS/PERM and 2 patients with focal epilepsy with GlyR aAb found by routine screening. The clinical and pathomechanistic data of all patients, except Patient 8, have been described in previous studies (Rauschenberger et al., 2020; Rauschenberger et al., 2023; Wiessler et al., 2024b; Wiessler et al., 2025).

Patient 8 (female, 42 years) had developed stiffness and dysarthria over the last 2 years, 6 months after giving birth and 2 weeks after infection. Upon examination, she had severe dysarthria and dysphagia, was wheelchair-bound, and needed help in most everyday tasks. GlyR aAbs were 1:32 in the serum but negative in the CSF. Under the diagnosis of PERM, the patient underwent plasmapheresis, which mildly improved leg tonus and swallowing.

### 2.3 Sequence alignments and homology modeling

Sequence alignments were created using GlyR subunit FASTA files (GlyRα1 human, UniProt P23415-1; GlyRα3L human, UniProt O75311-1; GlyRα1 *danio rerio* NCBI NP_571477.1) and the T-COFFEE web server (version 11.00) (Notredame et al., 2000; Di Tommaso et al., 2011). Modeling of GlyRα1 and GlyRα3 residue exchanges was performed in PyMOL (DeLano Scientific, San Carlos, CA, USA) based on the recent cryo-EM structure of heteropentameric GlyR (PDB: 7MLY; Zhu and Gouaux, 2021).

### 2.4 Cloning

The GlyRα1 and GlyRα3 variants were produced by exchanging residues between α1 and α3 using overlap extension site-directed mutagenesis. As a template, the human wild-type full-length GlyRα1^WT^ or GlyRα3^WT^L (L = long splice variant including the motif ^325^TEAFALEKFYRFSDM^339^ in the intracellular loop of GlyRα3; residues refer to the mature protein) cDNA in the pRK5 vector was used. The primers used for mutagenesis are listed in Supplementary Table 1. We used 100 ng/μl template DNA, 10 pmol/μl sense and antisense primers, 10 mM dNTPs, 10x Pfu buffer with BSA, and Pfu polymerase to amplify overlapping amplimers containing the mutated sequences. In a second overlap-PCR, the two amplimers were elongated at the 3′ ends by adding 10 mM dNTPs, 10x Pfu buffer with BSA, and Pfu polymerase with the following PCR conditions: Parental primers were added (10 pmol/μl), and PCR continued with the subsequent conditions. The PCR products and appropriate vectors (GlyRα1^WT^ or GlyRα1^WT^L) were digested with restriction endonucleases. Ligated plasmid DNA was transformed into competent *Escherichia coli* DH5α cells. All variants were verified using sequencing (Eurofins Genomics Germany GmbH, Ebersberg, Germany).

### 2.5 Cell line

For *in vitro* experiments, HEK-293 cells (Human Embryonic Kidney cells; CRL-1573; ATCC—Global Bioresource Center, Virginia, USA) were grown in minimum essential medium (Life Technologies, Massachusetts, USA). The medium was supplemented with 10% fetal bovine serum, L-glutamine (2 mM), 100 U/mL penicillin, and 100 μg/mL streptomycin at 37 °C and 5% CO_2_.

### 2.6 Cell transfection

HEK-293 cells were transiently transfected using calcium phosphate precipitation. A total of 200,000 cells were seeded on glass coverslips in a 35 mm cell culture dish and transfected after 24 h. GlyRα1, GlyRα3, and different variants of both subunits (Table 1) were co-transfected with eGFP to control the transfection efficiency (1 μg of each plasmid DNA). DNAs were supplemented with 2.5 M CaCl_2_, 0.1x TE buffer, and 2x HBS buffer (50 mM HEPES, 12 mM glucose, 10 mM KCl, 280 mM NaCl, 1.5 mM Na_2_HPO_4_). After 20 min of incubation at room temperature (RT, ~21 °C), the mix was applied to the cells. The medium was exchanged after 4–6 h. Cells were used for experiments 48–72 h after transfection.

**Table 1:** GlyRα1/α3 constructs.

Variant	Construct
GlyRα1	GlyRα1^WT^
Variant 1	GlyRα1^I132L, A137S^
Variant 2	GlyRα1^I132L, A137S, E173D, Q174E, G175A, A176P^
Variant 3	GlyRα1^A4R, P5S, K6A^
Variant 4	GlyRα1^N76S^
Variant 5	GlyRα1^H107N^
Variant 6	GlyRα1^S121F, R122K^
Variant 7	GlyRα1^A212V^
GlyRα3	GlyRα3L^WT^
Variant 8	GlyRα3L^L132I, S137A^
Variant 9	GlyRα3L^L132I, S137A, D173E, E174Q, A175G, P176A^
Variant 10	GlyRα3L^R4A, S5P, A6K^

### 2.7 Immunocytochemistry

For live-cell staining, transfected cells were incubated with patient sera diluted 1:50 or a commercial monoclonal antibody against GlyR*α*1 (mAb2b, mouse: 146111 or rabbit: 146118, Synaptic Systems, Göttingen, Germany) diluted 1:500 in cell medium for 2 h. After fixation for 10 min using 4% paraformaldehyde (PFA) with 4% sucrose in phosphate-buffered saline (PBS; pH 7.4), the cells were blocked with 5% goat serum in PBS for 30 min. If permeabilization of cells were required, blocking was performed with 5% goat serum and 0.2% Triton-X-100 in PBS. The primary antibody mAb4a, which is a pan-α GlyR antibody (mAb4a, 146,011, Synaptic Systems), was used at a dilution of 1:250 in blocking solution for 1 h at RT. Secondary antibodies goat-anti-human-IgG-Cy3 (109-165-003, Dianova, Hamburg, Germany), goat-anti-mouse-Cy3 (115-165-003, Dianova, Hamburg, Germany), or goat-anti-rabbit-Cy3 (111–165-003, Dianova, Hamburg, Germany) were diluted 1:500 in blocking solution and incubated with the cells for 1 h at RT in the dark. Cell nuclei were stained with 4′,6-diamidino-2-phenylindole (DAPI) 1:5,000 in PBS for 5 min and mounted on microscope slides with Mowiol.

### 2.8 Solid phase peptide synthesis

Peptides were synthesized using PurePep Chorus (Gyros Protein Technologies, USA) with Fmoc chemistry at a 0.05 mmol scale. Tentagel S RAM (Resin Substitution 0.22 mmol/g - Rapp Polymere, Germany) resin was first swollen in dimethylformamide (DMF) for 30–60 min. Next, Fmoc was removed using a 20% piperidine in DMF solution for 1–2 min and washed 3x with DMF. Deprotection steps were performed at 90 °C for the unphosphorylated peptides and at room temperature from the first phosphoserine coupling. The peptide chain was elongated by adding a premixed amino acid (AA, 125 mM and Oxyma, 125 mM) along with N, N′-diisopropylcarbodiimide (DIC, 250 mM). The mixture was heated at 90 °C for 2 min and then washed 2x with DMF. Capping was performed with acetic anhydride (10% in DMF) for 5 min and washed 3x with DMF. The peptide chain was elongated until completion and dried for 30 min after 7x DCM washes. The Fmoc-deprotected peptides were cleaved from the resin using a mixture of 90% TFA, 5% water, 3% TIPS, and 2% DCM for 4 h at RT. The peptides were then precipitated in ice-cold ether ON; purified using HPLC; and analyzed by LC–MS, as described below.

### 2.9 Peptide purification

The crude Peptides were purified by reverse-phase (RP) HPLC using a water-acetonitrile gradient with 0.1% formic acid (FA). Preparative HPLC was performed on an UltiMate™ 3,000 HPLC System (Thermo Fisher Scientific, Waltham, Massachusetts, USA) equipped with an RS variable wavelength detector (set to 215 nm) and a Kinetex® 5 μm EVO C18 100 LC column (00D-4633-P0-AX, Phenomenex, Aschaffenburg, Germany). Chromatograms were recorded using Chromeleon software v7.2. The purity and structural identity of the peptides were verified using a DAD-equipped 1,260 Infinity II HPLC device with a C18 RP column (Onyx Monolithic C18 50 × 2 mm), coupled to a mass selective detector with a single quadrupole system (Agilent Technologies, Santa Clara, CA, USA) in ESI + mode.

### 2.10 Neutralization assay

For neutralization of Patient aAbs, peptides as positive binding sequences were used. A “scrambled” (scr) peptide with a randomly distributed order of amino acids served as a negative control. Patient sera or commercial antibody mAb4a (146,011, Synaptic Systems) were diluted 1:500 according to their titer, with the highest dilution that revealed clear binding in previous tests, and incubated with 0.2 mM of the respective peptide, a mixture of peptides, or the “scrambled” peptide in PBS pH 7.4 for 30 min at 300 rpm. Afterwards, transfected HEK-293 cells were stained under living conditions with pre-neutralized patient sera, similar to the use of primary antibodies described previously.

### 2.11 Image analysis

Images were taken with a confocal Olympus Fluoview ix1000 microscope (Olympus, Hamburg, Germany) using a UPLSAPO 60x oil objective. All images were captured in 1,024 × 1,024 pixels and 16-bit and processed for analysis using the Fiji/ImageJ software (Schindelin et al., 2012).

### 2.12 Experimental design

All experiments were performed at least three times.

## 3 Results

**Figure 1:**
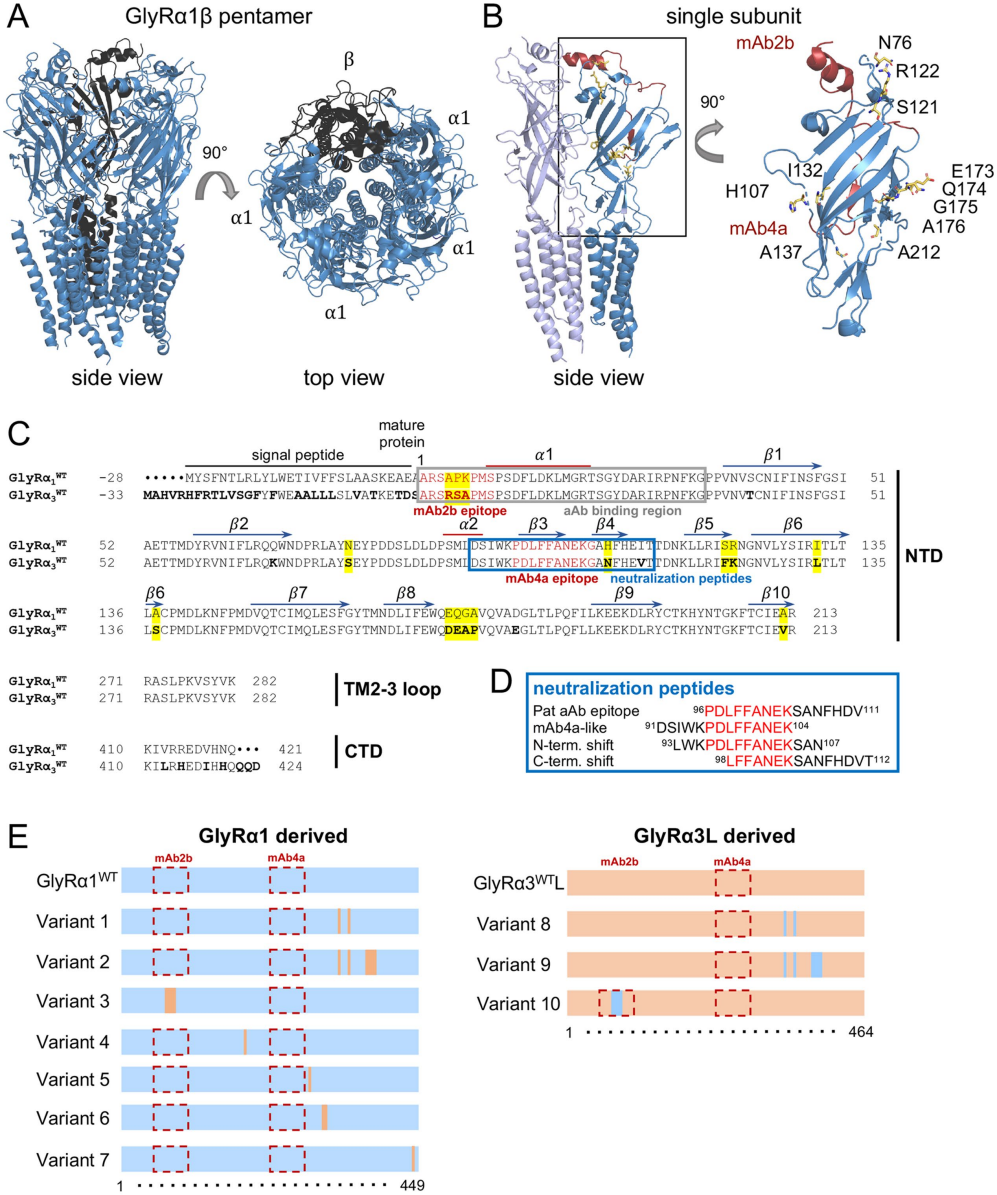
Overview of GlyR*α*1/α3 constructs. **(A)** Overall structure of the heteromeric GlyR pentamer (side and top views), with α-subunits in blue and the *β* subunit in dark gray (PDB: 7MLY; Zhu and Gouaux, 2021). **(B)** The subunit interface between two GlyR α-subunits (indicated by different blue colors, light blue and blue) is depicted. Within the darker blue α-subunit (right subunit), the epitopes of commercial antibodies are labeled in red, and residues different between GlyRα1 and GlyRα3 are marked in yellow (as in **C**) and labeled by residue numbers in the enlarged extracellular domain (ECD) of a single subunit. **(C)** Sequence alignment of ECD of GlyRα1 (UniProt P23415-1) and GlyRα3L (UniProt O75311-1), including the N-terminal domain (NTD), transmembrane 2–3 loop (TM2-3 loop), and the C-terminal domain (CTD). Residues differing between GlyRα1 and GlyRα3 are displayed in bold letters, and missing residues in the alignment are marked by a dot. Residues or small domains exchanged between GlyRα1 and α3 are labeled with a yellow background. The commonly proposed aAb-binding epitope (Rauschenberger et al., 2020) is marked by a gray box. The blue box indicates the location of the neutralizing peptides used. The epitopes of the commercial antibodies, mAb2b (GlyRα1-specific) and mAb4a (pan-α), are depicted in red. Amino acid residue numbers refer to mature proteins. Secondary elements are labeled above the sequence, including two α-helices and 10 β-sheets. **(D)** Sequences of the neutralizing peptides: a mAb4a-like epitope identified in a patient with aAbs against GlyRβ; the mAb4a epitope N-terminally elongated; and the mAb4a N-terminally shortened combined with C-terminal elongation. **(E)** Scheme of GlyRα1/α3L Constructs. Exchanges of amino acids between GlyRα1 (blue) and GlyRα3 (orange) are marked, as well as existing epitopes for commercial antibodies mAb2b and mAb4a (red dotted box).

GlyR aAb binding has mainly been demonstrated to the α1 subunit. However, for some patient sera, binding has also been described for GlyRα2, which is less common than α3, and GlyRβ, probably because of the high homology in the GlyR extracellular domain (ECD) (Figure 1) (Carvajal-Gonzalez et al., 2014; Rauschenberger et al., 2020; Rauschenberger et al., 2023; Wiessler et al., 2024a). These findings indicate that, in addition to the identified common epitope at the far N-terminal portion of GlyRα1, other antibody epitopes possibly exist within the ECD sequence (Rauschenberger et al., 2020).

### 3.1 Distinct patient aAb binding epitopes within GlyRα1/α3 variants

The pentameric native GlyR is presented by αβ heteromers with a subunit composition of 4α to 1β (Figure 1A) (Yu et al., 2021; Zhu and Gouaux, 2021). The GlyR ECDs encompass the N-terminal domain (NTD), which includes two α-helical elements and 10β-sheets; the small loop between transmembrane domains 2 and 3 (TM2-3 loop), and the C-terminal domain (CTD), and harbor individual residues that differ between the GlyRα1 and α3 subunits (Figures 1B,C). Patient sera were tested for specific binding to different GlyRα subunits (Wiessler et al., 2024b). All patient sera used in this study bound to GlyRα1 but not to GlyRα3 (only Patient 2 showed minor binding). The human GlyRα3 exists in two splice isoforms, which differ in 15 amino acids (the long isoform includes 15 additional amino acids (^225^T-^339^M); the short isoform (K) lacks the alternative splice cassette) in the large intracellular loop between transmembrane domains 3 and 4 (Nikolic et al., 1998). As the aAbs bind to the extracellular GlyR domain, the splice isoform was rather dispensable. We used the long (L) isoform GlyRα3L for mutagenesis. In addition to GlyRα1^WT^ and GlyRα3L^WT^, 10 NTD variants of GlyRα1 or GlyRα3L were established and used to transfect HEK-293 cells for immunostaining with patient aAbs (Figure 1E; Table 1). Variants 1 to 7 were derived from GlyRα1, whereas GlyRα3 was the origin of variants 8 to 10. Variants 1 and 8 had two corresponding exchanges at the C-terminal end within the NTD (^132^I in *α*1/^138^L in α3 and ^137^A in α1/ ^143^S in α3, residues refer to the mature protein). Variants 2 and 9 present extended amino acid exchanges of variants 1 and 8 (^132^I, ^137^A, ^173^E, ^174^Q, ^175^G, and ^176^A in α1 exchanged with ^132^L, ^137^S, ^173^D, ^174^E, ^175^A, and ^176^P in α3). Variants 3 and 10 contain three amino acid exchanges within the common N-terminal aAb epitope, which also harbors the α1-specific antibody epitope of mAb2b. With these amino acid exchanges, the epitope for the commercial antibody mAb2b was generated in GlyRα3 and eliminated from GlyRα1 (Figure 1E). Variants 4 (GlyRα1^N76S^), 5 (GlyRα1^H107N^), 6 (GlyRα1^S121F, R122K^), and 7 (GlyRα1^A212V^) displayed single or double amino acid exchanges in GlyRα1, with residues present at corresponding residues in α3.

**Figure 2:**
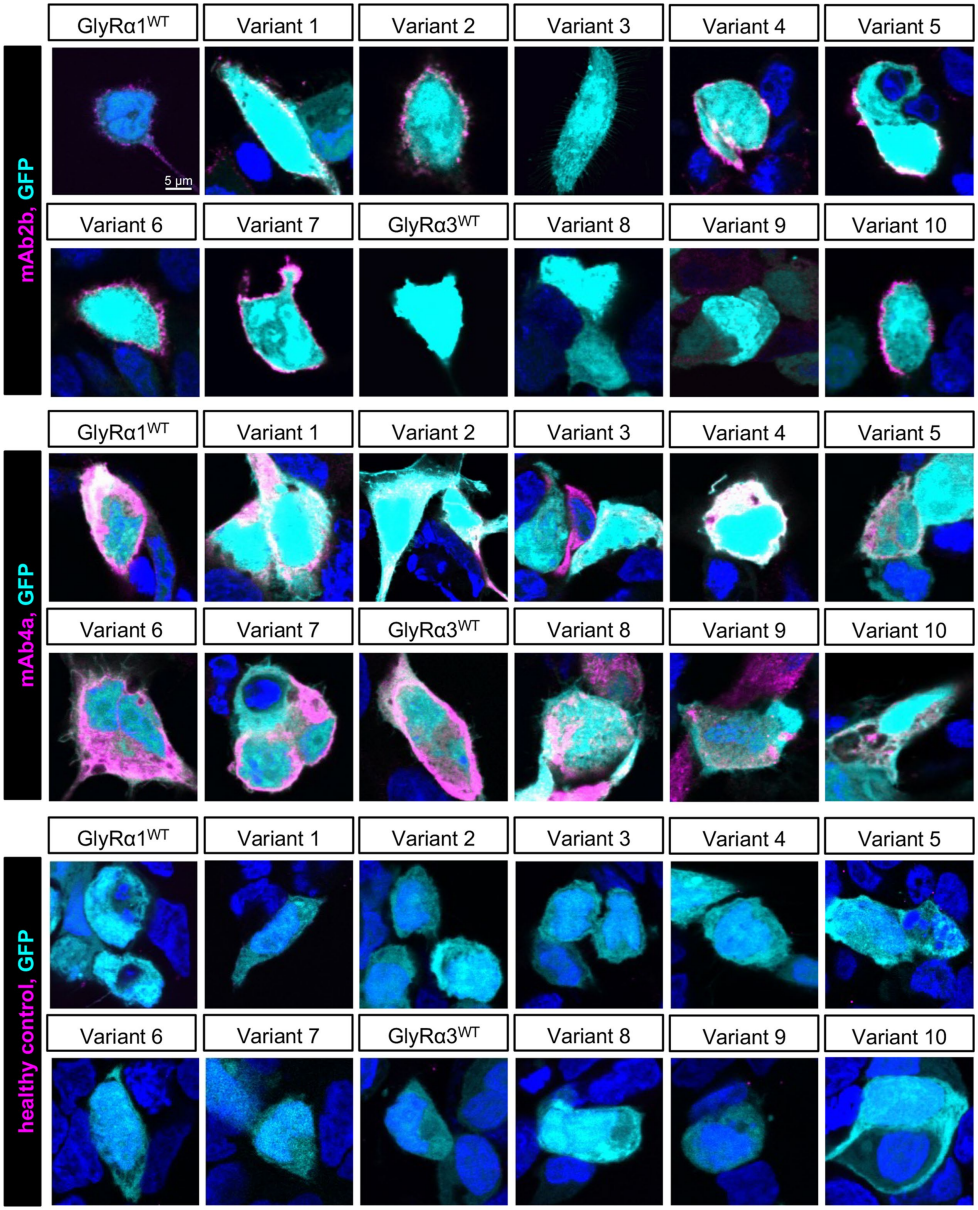
Expression of GlyRα1/α3 Variants. Immunocytochemical staining using commercial antibodies mAb2b (α1-specific) and mAb4a (pan-α) and healthy control serum (magenta) on HEK-293 cells expressing eGFP (cyan) and GlyRα1^WT^, GlyRα3^WT^L, and Variants 1–10. Cell nuclei are labeled with DAPI (blue). The scale bar indicates 5 μm.

To evaluate whether mutagenesis affects or impacts GlyR folding, transport, and expression levels of the GlyRα1 and GlyRα3 variants, the commercial antibodies mAb2b (α1-specific) and mAb4a (pan-α) were used to estimate receptor localization subsequent to transfection of the variants into HEK-293 cells. All variants were additionally subjected to healthy control serum that served as a negative control (Figure 2). The commercial antibody mAb2b only revealed specific binding to GlyRα1^WT^ and all its variants, preserving the mAb2b epitope and Variant 10, reflecting GlyRα3 with an included mAb2b epitope. The pan-α antibody mAb4a detected all variants, arguing that all mutant variants 1–10 are expressed in transfected HEK-293 cells. Healthy control serum did not reveal aAb binding to any expressed variant (Figure 2).

**Figure 3:**
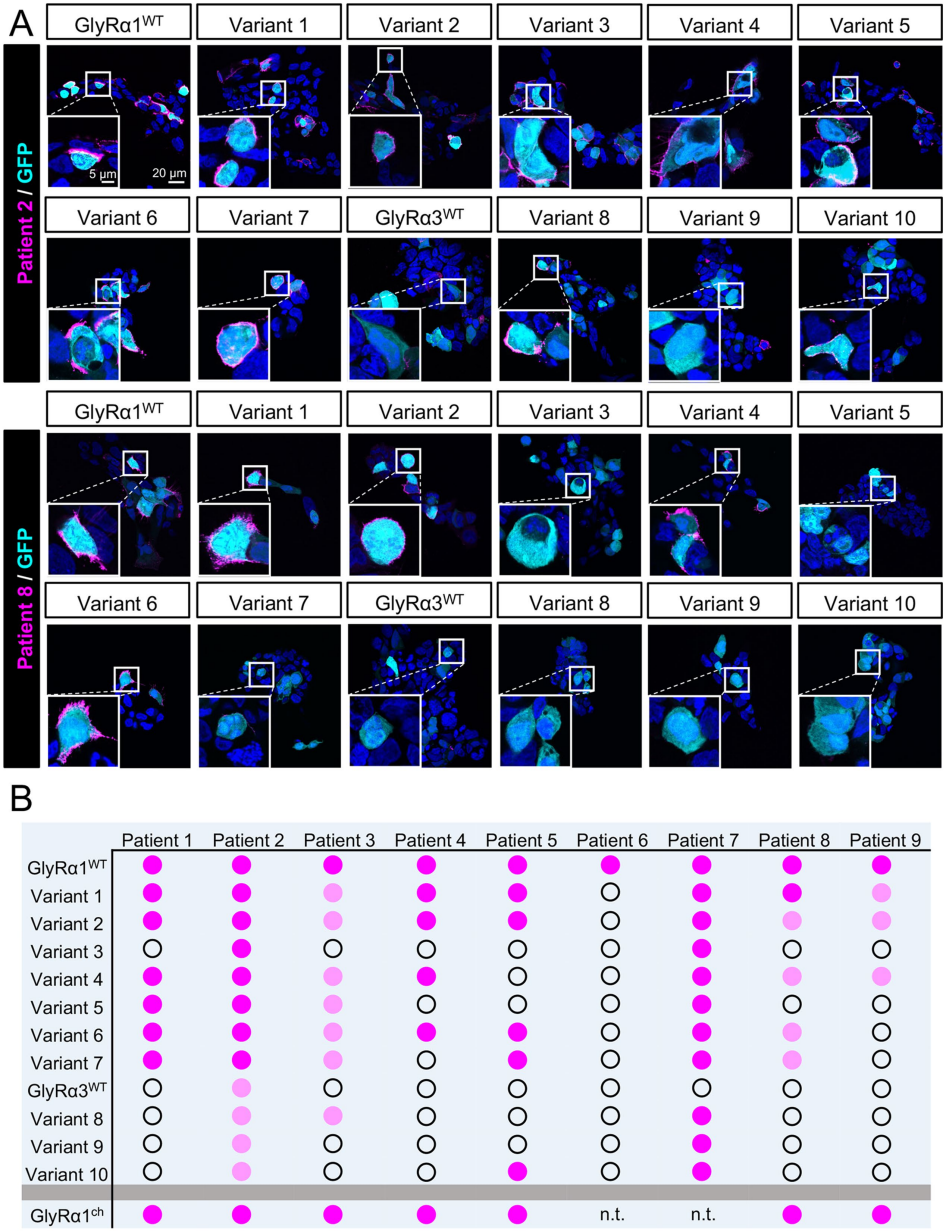
Patient-specific aAb epitope characterization using GlyRα1/α3 Constructs. **(A)** Immunocytochemical staining of aAb binding from Patients 2 and 8 (magenta) on HEK-293 cells expressing eGFP (cyan) and GlyRα1^WT^, or GlyRα3^WT^L and Variants 1–10. Cell nuclei are labeled with DAPI (blue). Scale bars indicate 20 μm (overview) or 5 μm (enlargement). **(B)** Binding properties of Patient aAbs to GlyRα1^WT^, GlyRα3^WT^L, and Variants (light magenta = weak binding, dark magenta = strong binding, open circle = no binding).

Nine patient sera samples positive for GlyR aAbs were tested for binding to the different GlyR*α*1/α3 constructs (Figure 3). Patients 2 and 8, who resemble very distinct binding patterns, are displayed with exemplary pictures (Figure 3A). The aAb binding in all individual stainings (N ≥ 3) was performed by five evaluators unaware of the identity of the slides. Scores were classified as positive (dark magenta) only when all experiments resulted in aAb binding for the majority of evaluators; negative (open circle) only when all experiments resulted in no aAb binding for the majority of evaluators; and +/− when there was just minor binding or not all evaluators classified the binding as positive (light magenta, Figure 3B).

Some patients showed distinct binding characteristics (Patients 4, 5, and 9), while others, e.g., Patients 2 and 7, exhibited binding to most variants. The binding patterns were similar for Patients 1, 3, and 8. The binding intensities were reduced for almost all modifications in GlyRα1 for Patients 3, 8, and 9.

The ^4^APK^6^➔^4^RSA^6^ exchange in the GlyRα1 variant 3 caused a loss of antibody binding for Patients 1, 3, 4, 5, 6, 8, and 9; exchanging them back in the GlyRα3 variant 10 only restored antibody binding for Patient 5. Therefore, ^4^APK^6^ introduction in GlyRα3, restoring a mAb2b epitope in GlyRα3, is not yet sufficient to restore aAb binding. The loss of binding to GlyRα1 lacking the mAb2b epitope suggests that these residues of the extracellular domain are necessary for aAb binding and part of the aAb epitope. Additionally, Abs from Patient 5 are sensitive to exchanges close to the pan-α mAb4a binding site (indicated by no binding on GlyRα1 variants 4 and 5), whereas further N-terminal exchanges do not impact binding behavior (GlyRα1 variants 1, 2, 6, 8, and 9, including residues ^121^S, ^122^R, ^132^I, ^137^A, and ^173^E-^176^A in GlyRα1).

Patients 2 and 7 revealed that aAb binding to each variant emerged from GlyRα1. While aAb binding on mutant GlyRα3 variants was not strong for Patient 2, Patient 7 was only negative for GlyRα3 and positive for all derived variants. These observations propose for those two patients that residue exchanges between GlyRα1 and GlyRα3 that were not covered by the generated mutant α1 or α3 variants, e.g., S40T or Q67K (Figure 1C), might be involved in aAb binding. Additionally, a combined number of exchanges found in the variants may be necessary to detect specific GlyRα1 or GlyRα3 aAb binding for these patients.

Binding pattern analysis for Patients 3, 6, 8, and 9 revealed that all exchanged positions in the N-terminal region of GlyRα1 played a role in aAb binding, as binding was abolished or very weak for almost all variants.

Overall, the binding pattern analyses identified individual residues that differ between GlyRα1 and GlyRα3 within the extracellular N-terminal domain that explain and underlie a specific aAb targeting GlyRα1 and GlyRα3 in this group of patients. Simultaneously, these findings indicate a role of conformational and/or bivalently bound epitopes of GlyR aAbs within the GlyR ECDs, as mutant variants of either GlyRα1 or GlyRα3 gave rather patient-specific but no clear common binding pattern. Additionally, the polyclonal composition of aAbs might also be a factor underlying the variable binding characteristics among patient sera.

### 3.2 GlyR H107 was identified as participating in an aAb binding epitope

**Figure 4:**
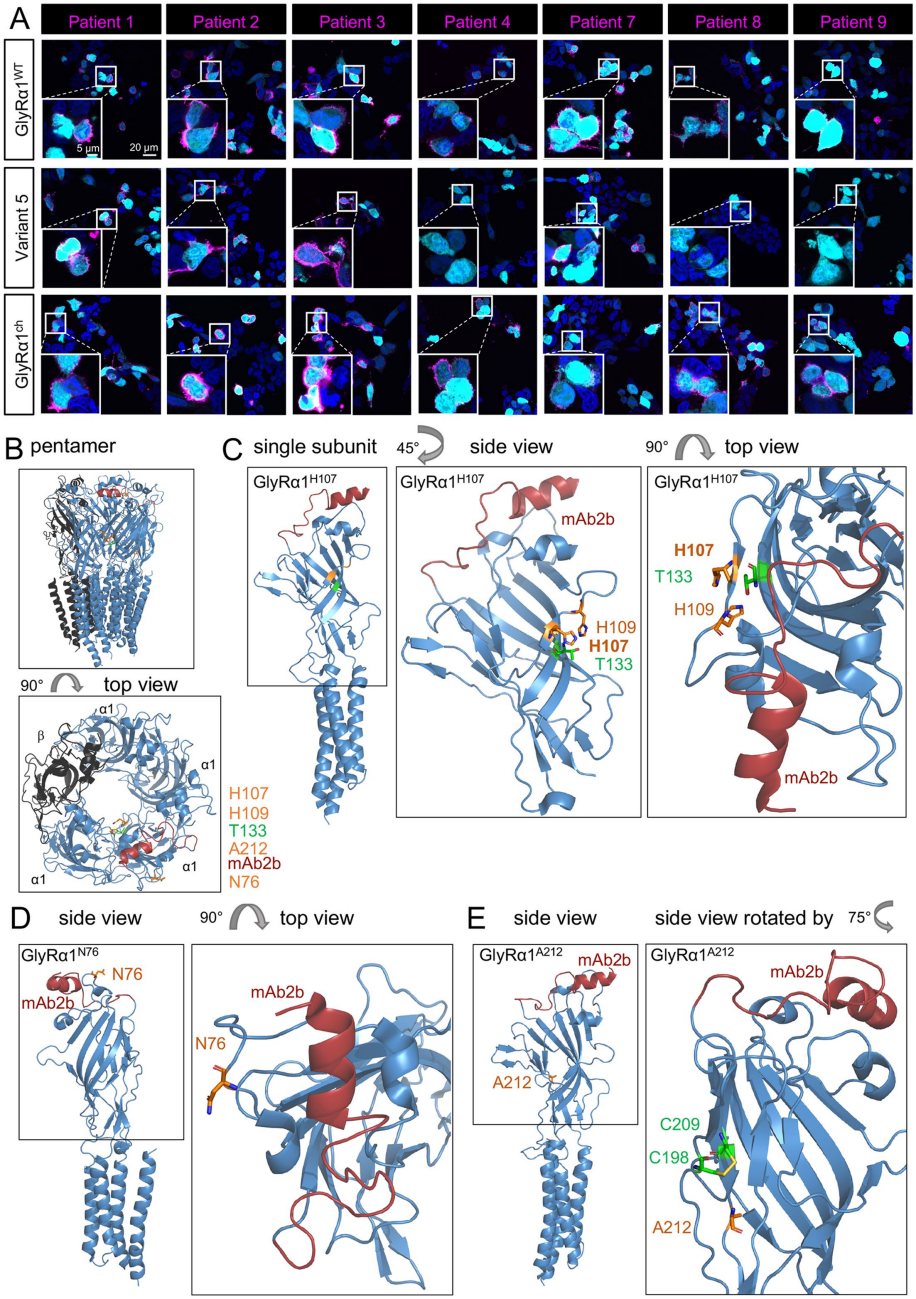
Histidine 107 in GlyRα1 plays an important role in aAb binding to an epitope that includes residue 107. **(A)** Immunocytochemical staining of Patient aAb (magenta) on HEK-293 cells transfected with eGFP (cyan) and either human GlyRα1, human GlyRα1^H107N,^ or zebrafish (*Danio rerio* = *dr*) GlyRα1*^dr^*. Cell nuclei are labeled with DAPI (blue). Scale bars refer to 20 μm and, in enlargements, to 5 μm. **(B)** Left: Pentameric structure (side view and top view) of GlyRα1β (based on the cryo-EM structure of the native heteromeric GlyRα1β structure from pig spinal cord; PDB: 7MLY; Zhu and Gouaux, 2021). The mAb2b binding epitope is marked in dark red, residues of interest are labeled in top view (orange and green). **(C)** One GlyRα1 subunit is displayed with the regions of interest H107 and H109 in orange and T133 in green. Enlarged and rotated regions of interest for better visualization. Right: Top view of the enlarged region of interest with marked residues. **(D)** Side and top views of one GlyRα1 subunit, looking at the subunit from inside the receptor extracellular lumen, are displayed with the region of interest N76 in orange. **(E)** Side views of one GlyRα1 subunit are displayed with A212 in orange, and C198 and C209, the two cysteine residues that are part of loop C, in green. Modeling was conducted using PyMOL.

To unravel the complex mechanisms underlying individual aAb binding, the impact of the exchanged residues required further investigation. Variant 5 harbors a single amino acid exchange, H107N (GlyRα1^H107N^), but strongly affects aAb binding. The binding of patient sera 4, 8, and 9 was disrupted if the mutant Variant GlyRα1^H107N^ was expressed in transfected HEK-293 cells (Figure 4A). H107 is located at the C-terminus of the mAb4a epitope. Histidine 107 in human GlyRα1 is not only an asparagine in human GlyRα3 but is also present in GlyRα1 of *Danio rerio* (*dr*, zebrafish). Although the ECD of GlyRα1^dr^ differs from that of human GlyRα1 in only five individual residues, most aAbs against the GlyRα1 bind to human but not the zebrafish GlyRα1 (Rauschenberger et al., 2020). Patients 5 and 6 were not tested here because of limited material availability from these patients. For Patients 1, 2, 3, and 7, aAb binding remained for GlyRα1^H107N^-expressing cells (Figure 4A). Next, we used a chimera (GlyRα1^ch^) of the human GlyRα1 N-terminus up to glycine 34 and the ECD from zebrafish GlyRα1 (GlyRα1^dr^) (Supplementary Figure 1) (Rauschenberger et al., 2020). This chimera carried an asparagine at position 107 and was able to restore aAbs binding from tested patients (Patients 4, 8, and 9). The observed lack of binding to human GlyR*α*1^H107N^ for Patient sera 4, 8, and 9 may therefore be due to a disrupted structural integrity of GlyRα1 at position H107N necessary for aAb binding of Patients 4, 8, and 9.

To further investigate whether residue 107 in the 3D structure interacts with or is closely located to the common aAb binding site in the N-terminal of GlyRα1, we used the heteromeric GlyR cryo-EM structure (PDB: 7MLY; Zhu and Gouaux, 2021). Histidine residue 107 is located in the short β4-sheet (Figures 1C,4B,C). Binding of aAbs to this region may impact the structural dynamics surrounding H107 in human GlyRα1. There is also evidence from aAbs binding to other Cys-loop receptors, such as acetylcholine receptors (AChRs), that residues in the vestibule of the ion channel can be targeted by IgGs (Li et al., 2025).

Additionally, the two single amino acid changes N76S (β2–3 loop in GlyRα1 structure) and A212V (β10 in GlyRα1 structure) revealed that these residues are crucial for aAbs binding, since mutations of these amino acids abolished the binding of aAb for Patients 5 and 6 to N76S and for Patients 4, 6, and 9 to A212V (Figures 4D,E).

These findings further demonstrate that GlyR aAbs not only bind to one additional common region within the ECD but also to multiple binding sites or additional domains that impact aAb binding are present. Hence, there is an essential need to evaluate the binding epitopes for each individual patient.

### 3.3 Small peptides resembling aAb epitopes can neutralize aAb binding to GlyRs *in vitro*

**Figure 5:**
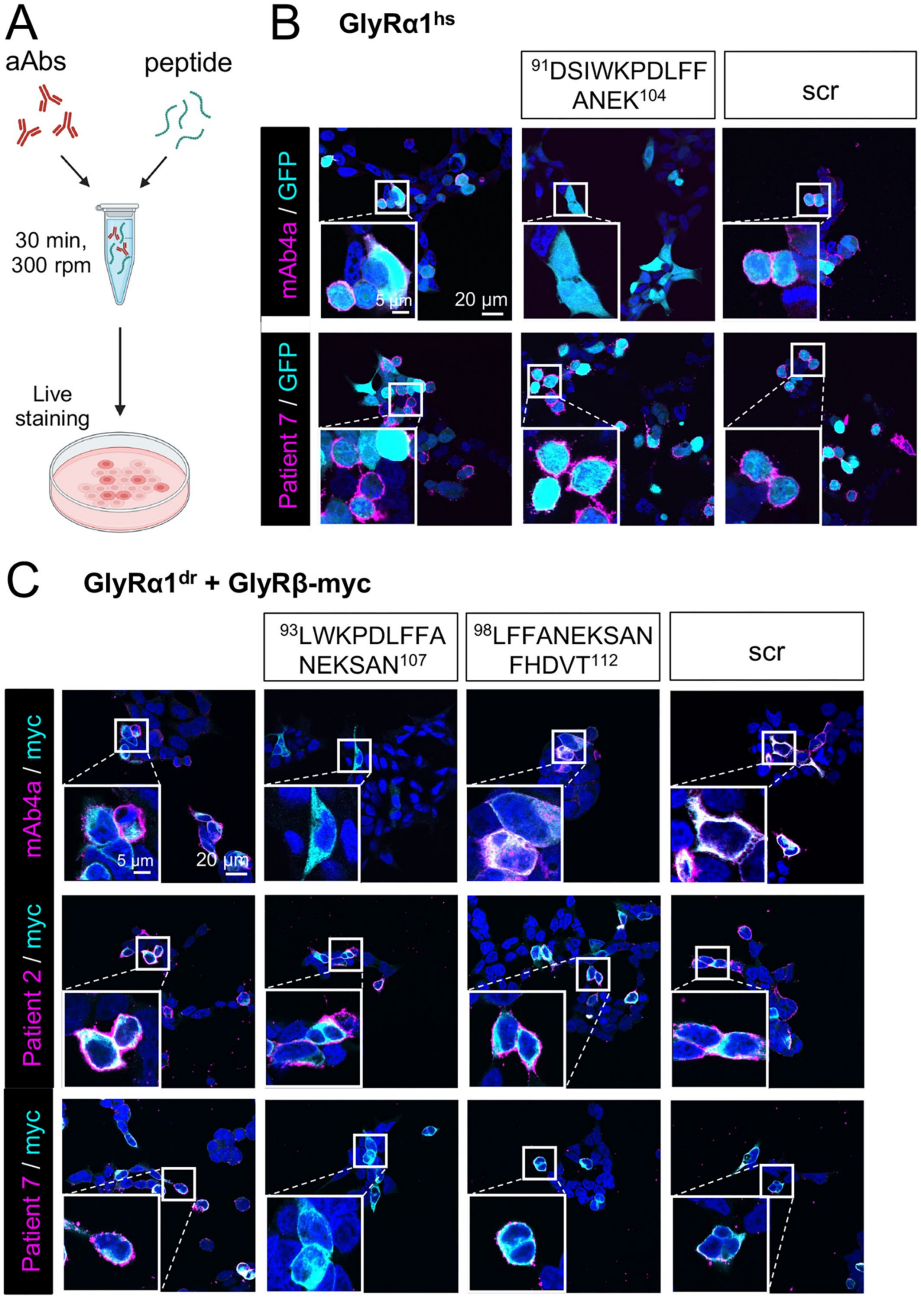
Peptides resembling a distinct sequence of aAb epitopes can eliminate binding to GlyRs. **(A)** Scheme of the neutralization assay using patient aAbs and purified peptides. **(B–C)** Immunocytochemical staining of (B) GlyRα1^hs^ and GFP (cyan) or **(C)** GlyRα1^dr^ and GlyRβ-myc (cyan) co-transfected HEK-293 cells together with patient aAbs or mAb4a staining (magenta). The commercial antibody mAb4a (upper panel) or Patient samples (lower panel) were pre-incubated with **(A)** the ^91^DSIWKPDLFFANEK^104^ peptide and a scrambled (scr) peptide or **(B)** the N-terminal elongated ^93^LWKPDLFFANEKSAN^107^ peptide, the C-terminal elongated ^98^LFFANEKSANFHDVT^112^ peptide, and a scr peptide for 30 min prior to cell staining. Nuclei are labeled with DAPI (blue). Scale bars indicate 20 μm (overview) or 5 μm (enlargement).

Peptides that enable the binding and neutralization of patient aAbs using microarray experiments (Wiessler et al., 2024b) were investigated with native GlyRs *in vitro* in transfected HEK-293 cells (Figure 5A). We used peptides resembling the pan-α mAb4a epitope as a common region between all GlyRα subunits (Figure 1C; Supplementary Figure 1), which has been previously shown to determine the binding of Patient 7 (Wiessler et al., 2024b). However, a peptide resembling the mAb4a binding epitope (^91^DSIWKPDLFFANEK^104^, Figure 1D) was not able to eliminate aAb binding to GlyRα1 for various tested patients, as shown exemplarily for Patient 7 (Figure 5B). This can be explained by the presence of polyclonal aAbs in patient sera. Polyclonal sera are not straightforward to neutralize, as single-binding epitopes of the aAb pool may be missed. As a proof of principle, we used the commercial antibody mAb4a as a control, which was neutralized by the abovementioned peptides, thus validating the neutralization method (Figure 5B).

Specific neutralization of patient aAbs is, however, possible when epitope sequences are available. Therefore, we used the unique binding of Patient 7 to the GlyRβ subunit via the ^96^PDLFFANEKSANFHDV^111^ epitope, which includes the mAb4a binding sequence (Figure 1D; Supplementary Figure 1) (Wiessler et al., 2024b). The GlyRβ subunit, however, cannot be expressed at the cellular membrane alone without the presence of a GlyRα subunit, enabling the transport of GlyRβ to the cellular surface. Moreover, the binding of Patient serum 7 to human GlyRα1 was avoided by co-transfection of HEK-293 cells with human GlyRβ in combination with zebrafish GlyRα1 (*Danio rerio,* GlyRα1^dr^). The zebrafish GlyRα1 ensures the correct expression and localization of heteromeric GlyRs and is not bound by the aAbs of Patient 7. Only Patient 2 showed binding to GlyRα1^dr,^ making GlyRα1^dr^ a pivotal tool for studying aAb binding. We used two different overlapping short peptides (N-terminal shifted = ^93^LWKPDLFFANEKSAN^107^, and C-terminal shifted = ^98^LFFANEKSANFHDVT^112^, Figure 1D; Supplementary Figure 1) that resemble either an N-terminal or a C-terminal prolonged and slightly modified mAb4a epitope to neutralize patient sera in a 30 min incubation step before adding the combined mixture to the cells. A scrambled peptide (scr = AEDQWEFILDNMTYGFSE) served as a negative control, which was unable to neutralize commercial antibody or patient aAb binding.

Neutralization of the commercial antibody mAb4a worked for peptide 1, but peptide 2 could not neutralize mAb4a staining, as it lacked the N-terminal residues ^96^P^97^D (Figure 5C). Next, we tested Patients 2 and 7 for neutralization. Patient 2 had various binding epitopes, as it bound almost all GlyRα1 and GlyRα3 variants (Figure 3) in the antigen pool to some extent, and therefore should not be neutralized, enabling us to test the specificity of the tool. Indeed, no neutralization of Patient 2 aAb binding was observed, whereas for Patient 7, aAbs binding was abolished because of neutralization by peptide 1 but not by peptide 2 (Figure 5C). This result again suggests that the lack of residue ^96^P^97^D in peptide 2 disables Patient 7 aAbs from binding and hence neutralization. Scr was unable to neutralize commercial antibody or patient aAb binding.

With these experiments, we were able to demonstrate that neutralization of patient aAbs with short peptides is possible if the specificity of the estimated peptides for target binding has been obtained by additional methods, e.g., microarrays or chimeric GlyR variants. However, every patient sample requires testing for binding epitopes first, and individual peptide mixtures for every patient’s serum must be developed and validated. A limitation is that structural epitopes cannot be identified by this method and, hence, not addressed by neutralization approaches.

## 4 Discussion

Plasma exchange is one of the most widely used therapeutic strategies for patients with GlyR aAbs (Pagano et al., 2014; Doppler et al., 2016; Hinson et al., 2018). However, this approach indiscriminately removes immunoglobulins, including protective antibodies, from circulation. Treatment optimization remains a pressing need to improve patient therapy and eventually achieve a faster return to normal function (Pineda, 1999; Guptill et al., 2016). A deeper understanding of GlyR aAb binding patterns may enable the development of more selective and personalized therapeutic approaches. Recently, we studied GlyR aAb in different CNS regions and correlated its binding properties with some clinical manifestations (Piro et al., 2025). However, it remains unclear which amino acid sequences of the different GlyR subunits are targeted in tissue sections by aAbs. A common N-terminal epitope in GlyRα1 has been identified for GlyR aAbs, whereas for patient samples positive for GlyRβ binding, other distinct epitopes have been obtained (Rauschenberger et al., 2020; Wiessler et al., 2024b). Although all GlyRs share high sequence homology in the extracellular N-terminal domain (Lynch, 2004), binding to GlyRα2 or GlyRα3 has rarely been found (Carvajal-Gonzalez et al., 2014; Wiessler et al., 2025). To investigate GlyR aAb binding properties in detail, we studied GlyRα1 and GlyRα3 N-terminal variants for patient-derived antibody binding *in vitro* in cell-based assays. Immunocytochemical analyses revealed heterogeneous binding profiles among patients. Some patient sera displayed high sensitivity to even single amino acid substitutions (e.g., N76S, H107N, and A212V). In general, GlyRα3 WT or GlyRα3 variants do not represent common targets of GlyR aAbs. Reciprocal amino acid exchanges between GlyRα1 and GlyRα3 rarely restored binding, indicating that antibody recognition is not confined to a single epitope but likely involves multiple regions within the ECD. Recently, a novel epitopic landscape for monoclonal aAbs against ECD of AChRs has been described, including multiple binding sites (Li et al., 2025).

Disruption of the far N-terminal common GlyR aAb binding site, including the epitope of the GlyRα1-specific mAb2b binding site, leads to a lack of serum binding in most patients, facilitating the correctness of a common GlyRα1 binding sequence (Rauschenberger et al., 2020). However, the introduction of the mAb2b epitope in GlyRα3 did not restore binding of patient serum samples, again arguing that the common GlyR aAb epitope includes mAb2b, but in addition, the surrounding sequence contributes to the GlyR aAb binding site.

To further evaluate the non-binding of aAb upon single amino acid exchanges between GlyR*α*1 and GlyRα3, the structural consequences of these amino acid variations were evaluated using the 3D structure based on cryo-EM of the GlyR (Zhu and Gouaux, 2021). The α1^H107N^ exchange is not located close to the determined far N-terminal common aAb binding site. However, residue H107 in the short β4 sheet (Zhu and Gouaux, 2021) was in proximity to the mAb4a antibody epitope. An expanded mAb4a-like binding epitope for GlyR aAbs targeting the GlyRβ subunit has also been previously reported (Wiessler et al., 2024b). H107 resides within the suggested epitope for a patient carrying GlyRα as well as GlyRβ aAbs; our findings confirm that a second epitope besides the common epitope in the far N-terminal region of GlyRα1 for aAb binding exists.

The GlyRα1^N76S^ exchange also impaired GlyR aAb binding. Residue N76 is structurally located in the β2–3 loop of the GlyR NTD close to the proposed common epitope at the surface of the GlyRα1 protein if viewed from the top. Genetic GlyRα1 variants in the β2–3 loop (W68C, D70N, and R72H) associated with startle disease displayed very low abundance at the cellular surface, associated with the non-functionality of the mutated ion channels (Schaefer et al., 2015). Hence, this region is highly sensitive to amino acid exchanges with α1^N76S^, which most likely disrupts the integrity of the common GlyRα1 aAb binding epitope.

aAb binding is also altered by variant 7 (GlyRα1^A212V^). Alanine 212, located in β-sheet 10 of the GlyR NTD, is far away from the common far N-terminal aAb epitope but is located in loop C of the GlyR (Grutter et al., 2005; Vogel et al., 2009). Loop C is involved in neurotransmitter binding by capping the glycine binding pocket. Indeed, in other diseases associated with aAbs that target other types of Cys-loop receptors, such as *myasthenia gravis* (aAbs against AChR) and autoimmune encephalitis (autoantibodies against the GABA_A_ receptor), it has been shown that the aAbs target the neurotransmitter binding pocket (Noviello et al., 2022; Li et al., 2025). Hence, from our data, there is evidence that residue A212 is most likely directly involved in aAb binding.

In addition to the site-directed mutagenesis studies on GlyR variants and structural modeling to explain the lack of aAb binding, peptide microarrays have been used to map aAb epitopes, which were successfully shown for aAbs against Kv1.2, Contactin1, and a specific GlyR aAb against GlyRβ (Talucci et al., 2024; Wiessler et al., 2024b; Grüner et al., 2025). The extracellular region around the pan-α mAb4a epitope present in all GlyRα and β subunits has been previously determined to present a GlyR aAb epitope (Wiessler et al., 2024b). The use of the ^91^DSIWKPDLFFANEK^104^ peptide lacking the 3′ serine 105 of the mAb4a binding sequence was, however, unable to neutralize patient aAbs other than the monoclonal antibody mAb4a.

Lack of neutralization might be explained by (i) aAbs derived from polyclonal B cells that excessively generate these antibodies upon activation (Ishigatsubo et al., 1988; He et al., 2004; Stogerer and Stager, 2020). The outcome is a mixture of polyclonal antibodies that are not all directed against the same epitope of their target protein and therefore cannot all be eliminated upon incubation with the same peptide, which seems to apply for GlyR aAbs; (ii) the lack of a three-dimensional structure of the used peptides, which are linear amino acid sequences with a short length; (iii) aAb binding sites, which can also consist of discontinuous sequences that form a three-dimensional epitope that would neither be detected by peptide microarrays nor neutralized by the used approach (Schreiber et al., 2005; Katz et al., 2011; Sokolove et al., 2012; Sinmaz et al., 2016). Only in the folded protein, discontinuous sequences will spatially align and form an aAb binding epitope.

In Wiessler et al. (2024b) a specific peptide sequence including the mAb4a epitope for patient serum that targets the GlyRβ subunit was identified by high-resolution peptide microarray screening. Using the identified peptide sequence, ^91^DSIWKPDLFFANEKG^105,^ for neutralization of the patient’s aAbs abolished their binding to the peptides spotted on the microarray. Here, the use of two overlapping sequences in the GlyR sequence region, always including the full-mAb4a epitope but elongated either N- or C-terminal (^93^LWKPDLFFANEKSAN^107^, ^98^LFFANEKSANFHDVT^112^), revealed specific neutralization of GlyRβ aAbs by ^93^LWKPDLFFANEKSAN^107^, but not by ^98^LFFANEKSANFHDVT^112,^ which lacks the N-terminal residues proline 96 and aspartate 97. Hence, residues proline 96 and aspartate 97 are essential for correct epitope formation of the GlyRβ-specific aAbs. Using the same approach for other patient sera sometimes resulted in a reduction of binding but never in complete aAb neutralization. Thus, although neutralization experiments provide additional information on aAb binding domains for GlyR-positive patients, the presence of more than one binding epitope for patient aAb requires an extension of these types of experiments and the use of combined approaches. First, all epitopes within a serum sample must be identified to use an individual peptide mixture for each patient sample to eliminate aAb binding on living cells. Residual or domain exchanges in the target protein, combined with cell-based assays, are one step in identifying important regions for aAb binding (Rauschenberger et al., 2020; Rauschenberger et al., 2023). The use of linear overlapping peptide libraries on microarrays is another approach for testing aAb binding, which can be further confirmed by high-resolution microarray screens using peptides with more overlap and extension shifts of only one residue. Such approaches allow the clustering of patients sharing the same epitope but also determine inter-individual differences within a patient cohort (Talucci and Maric, 2024). Based on such combined methods, defined pools of peptide mixtures can be used to elucidate which peptides facilitate antibody binding and hence present a step forward for developing more personalized plasmapheresis-like therapeutic options. However, the presence of structural or discontinuous epitopes cannot be taken into account.

In summary, the polyclonality of GlyR aAbs renders aAb neutralization a great challenge. The generation of monoclonal antibodies from CSF samples from patients with autoimmune encephalitis (Kreye et al., 2016; Kornau et al., 2020; Ramberger et al., 2020) has provided precise mechanistic insights into the underlying pathological mechanisms. So far, its application to GlyR autoimmunity has failed, most likely because GlyR aAbs are often absent from CSF. Furthermore, because pathogenicity may arise from the collective action of polyclonal aAbs rather than from individual clones, serum-based analyses may still provide a more physiologically relevant perspective.

In conclusion, the identification of disease-specific GlyR aAb epitopes in neurological diseases, such as SPS/PERM, will enhance our current understanding of the pathological mechanisms, help contribute to antigen-specific treatments, and, hence, develop more efficient patient-specific therapies.

## Data Availability

The original contributions presented in the study are included in the article/Supplementary material, further inquiries can be directed to the corresponding author.
