# Supplementary material for: Polyclonal glycine receptor aAbs: a challenge for personalized epitope characterization
Source: Front Mol Neurosci. 2026 Mar 17;19:1747209. doi: 10.3389/fnmol.2026.1747209 (PMC13036184; doi:10.3389/fnmol.2026.1747209)
Supplement: Supplementary file 1 [file Data_Sheet_1.pdf]

Supplementary Material

1 Supplementary Figures and Tables

Supplementary Table 1: Primers for mutagenesis of GlyR Variants (produced by Life Technologies, Darmstadt, Germany).

Variant	*Forward Primer*	*Reverse Primer*
Variant 1	5’ C ATC AGA CTC ACC CTG ACA CTG TCC TGC CCA ATG GAC T 3’	5’ CAT TGG GCA GGA CAG TGT CAG GGT GAG TCT GAT GCT 3’
Variant 2	5’ GG CAG GAC GAG GCA CCG GTG CAG CTA GCA 3’	5’ C TAG CTG CAC CGG TGC CTC GTC CTG CCA 3’
Variant 3	5’ GCT GCT AGA TCT GCA AGT GCT CCT ATG TCA 3’	5’ CAT AGG AGC ACT TCG AGA TCT AGC AGC TTC 3’
Variant 4	5’ G AAC GAC CCG CGG CTG GCC TAT AGT GAA TAC CCT 3’	5’ C AGG GTA TTC ACT ATA GGC CAG CCG CGG GTC GTT CCA 3’
Variant 5	5’ G AAG GGG GCT AAC TTC CAT GAG ATC ACC ACA 3’	5’ GGT GAT CTC ATG GAA CTT AGC CCC CTT CTC GTT 3’
Variant 6	5’ C AAA TTG CTA AGA ATA TTC AAG AAT GGG AAT 3’	5’ GAC ATT CCC ATT CTT GAA TAT TCT TAG CAA TTT 3’

Variant 7	5’ GGT AAA TTC ACA TGC ATT GAG GTC CGG TTC CAC CT 3’	5’ CAG GTG GAA CCA GAC CTC AAT GCA TGT GAA TTT A 3’
Variant 8	5’ CA ATA AGA ATA ACA TTA ACA CTT GCC TGT CCC ATG GAT CTC A 3’	5’ GAG ATC CAT GGG ACA GGC AAG TGT TAA TGT TAT TCT TAT 3’
Variant 9	5’ GAA TGG CAA GAG CAG GGC GCC GTA CAA GTG GCA 3’	5’ C CAC TTG TAC GGC GCC CTG CTC TTG CCA TTC A 3’
Variant 10	5’ GAC AGT GCA AGG TCC GCA CCT AAG CCA ATG TCA 3’	5’ CAT TGG CTT AGG TGC GGA CCT TGC ACT GTC TGT 3’

Supplementary Figure 1

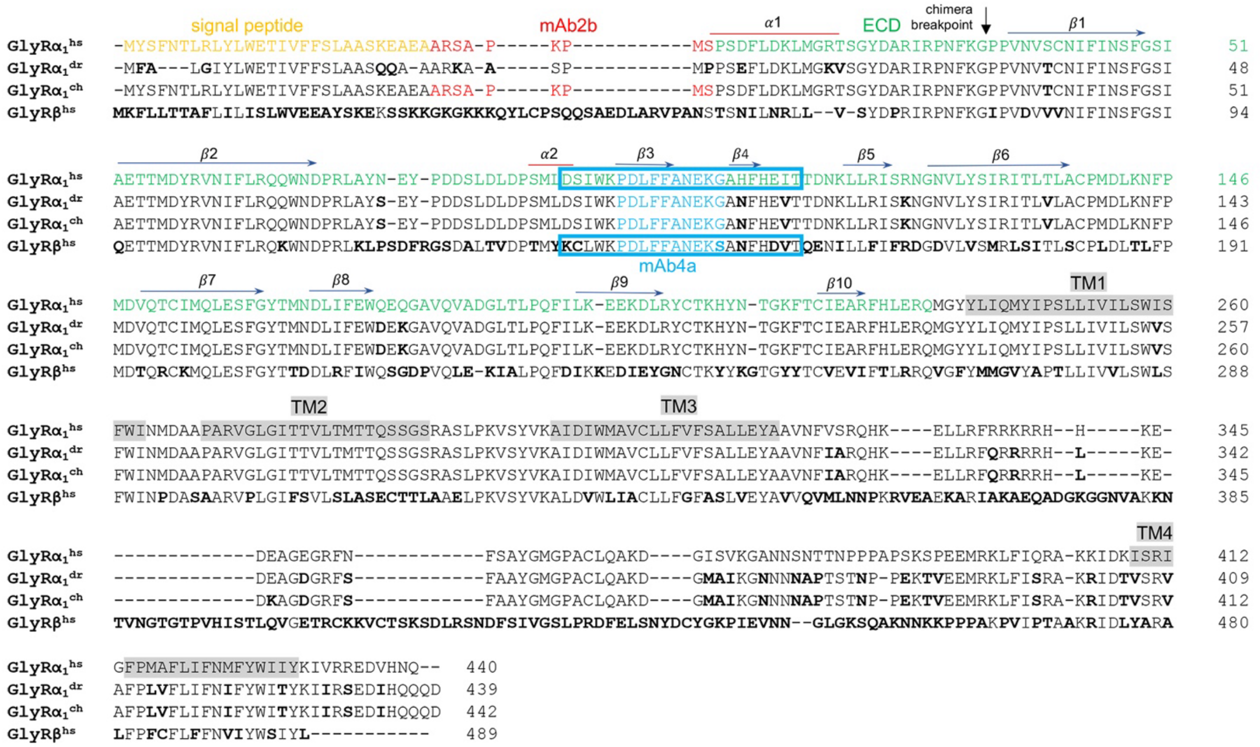

Supplementary Figure 1

Sequence alignment of human and zebrafish glycine receptor alpha 1 subunits (UniProt P23415-1, GlyRα1^{hs}; NP_571477.1, GlyRα1^{dr}), a chimera (GlyRα1^{ch}) generated from those sequences switching the N-terminal part, and the human GlyRβ subunit. The chimera breakpoint is at residue ^{34}G. Marked are the signal peptide of 28 amino acids in yellow, the residue 1 of the mature protein, the extracellular domain in green. In red and blue, the amino acid sequence targeted by commercial antibodies mAb2b and mAb4a are labeled. The blue box marks the region used for peptide generation to neutralize the GlyR aAbs. Note, while the sequence homology between GlyRα and GlyRβ is less pronounced, both sequences share the mAb4a epitope except for the last residue. Secondary structural elements are depicted above the sequence including two α-helices and 10 β-sheets, transmembrane domains TM are marked in grey.
